# Macrophage TMEM175‐Mediated Transcriptional Regulation of Cathepsins Drives Bacterial Killing in Sepsis

**DOI:** 10.1155/jimr/2046607

**Published:** 2026-09-18

**Authors:** Zhenzhen Bai, Bei Peng, Tao Luo, Lingbin Sun

**Affiliations:** ^1^ Department of Anesthesia, Peking University Shenzhen Hospital, Shenzhen 518036, China, pkuszh.com

**Keywords:** cathepsins, sepsis, TMEM175, transcriptional regulation

## Abstract

Sepsis is a life‐threatening condition characterized by a dysregulated host response to infection, in which lysosomal acidification and hydrolytic activity are crucial for effective pathogen clearance by macrophages. Transmembrane Protein 175 (TMEM175) is a key regulator of lysosomal pH, and its deficiency is reported to cause lysosomal over‐acidification and impaired cathepsin D (CTSD) activity. However, the role of TMEM175 in sepsis remains uncertain. We hypothesize that TMEM175 deficiency exacerbates sepsis by impairing CTSD activity. TMEM175 mRNA expression levels were measured in peripheral blood mononuclear cells (PBMCs) from septic patients and healthy controls (HCs). Using a cecal ligation and puncture (CLP) mouse model, we evaluated the effects of the TMEM175 inhibitor 2‐phenylcyclopentylamine (2‐PPA) on mortality and bacterial load in target organs. In vitro, TMEM175‐knockdown in macrophages was performed to assess its impact on bacterial clearance, phagocytosis, lysosomal acidification, and cathepsin maturation. Results showed that TMEM175 expression was significantly downregulated in PBMCs from septic patients. Pharmacological inhibition of TMEM175 in CLP mice exacerbated bacterial burden and increased mortality. Mechanistically, TMEM175 deficiency impaired macrophage‐mediated bacterial clearance, despite having no effect on the initial phagocytic uptake. This dysfunction was attributed to TMEM175, which directly regulates cathepsin transcription. Additionally, TMEM175 deficiency suppressed PI3K–Akt signaling, reducing inflammatory cytokine production.

## 1. Introduction

Sepsis is defined as a life‐threatening organ dysfunction caused by a dysregulated host response to infection and is an important global health problem [1]. Sepsis and septic shock are major healthcare problems, with epidemiological analyses estimating 48.9 million global cases and 11 million deaths in 2017, representing 19.7% of all deaths that year [2, 3]. The clearance of pathogenic microorganisms is the key factor affecting the prognosis of sepsis [4–6]. Autopsy reports point out, for sepsis in deceased patients, that most patients displayed unresolved septic foci, which shows that infection control failed in these patients [6, 7].

The innate immune system’s primary defense against bacterial infection is phagocytosis by macrophages, which engulf pathogens into phagosomes [8–10]. This process internalizes pathogens into cytoplasmic vacuoles, known as phagosomes [11, 12]. Subsequently, these newly formed phagosomes undergo a coordinated series of maturation events: they fuse with endosomes and lysosomes, triggering acidification, enhanced oxidative stress, and the enrichment of degradative enzymes [13]. Through the maturation cascade, phagosomes ultimately transform into phagolysosomes, the specialized compartments responsible for bacterial elimination [10, 13]. In this process, efficient bacterial clearance depends on phagosome/lysosome acidification and enhanced lysosomal enzyme activity. As lysosomal aspartic proteases, cathepsins are vital for killing pathogens and modulating immune responses, which require a highly acidic environment for optimal function [14–18]. The activity and localization of cathepsins, whether within lysosomes or translocated into the cytosol and extracellular space, are closely tied to lower lysosomal pH, highlighting the importance of acidification in innate immunity [19].

In certain situations, lysosomal acidification does not invariably lead to enhanced cathepsin activity. TMEM175, a crucial proton channel in lysosomal membranes, plays a pivotal role in the selective efflux of protons from the lysosomal lumen. The importance of TMEM175 has been highlighted in neurodegenerative diseases, where its loss results in lysosomal hyper‐acidification, impairs cathepsin function, and thus promotes α‐synuclein aggregation [20, 21]. Although TMEM175’s role in neurodegenerative diseases has been extensively studied [20–24], the inconsistency between lysosomal acidity and cathepsin activity remains largely unexplored.

Given the pivotal role of lysosomal acidification in macrophage bactericidal activity [4, 6], we hypothesize that TMEM175 influences cathepsin D (CTSD) activity by regulating lysosomal pH, which in turn affects the ability of macrophages to eliminate pathogens and modulate immune responses. Consequently, enhancing TMEM175 function may improve sepsis prognosis by restoring CTSD activity. To test this hypothesis, our study systematically investigated the role and underlying mechanisms of TMEM175 in macrophage antibacterial responses during sepsis. Initially, TMEM175 expression levels in peripheral blood mononuclear cells (PBMCs) from sepsis patients were assessed to establish clinical relevance. Subsequently, the cecal ligation and puncture (CLP) mouse model was employed to evaluate the impact of adoptive transfer of TMEM175‐expressing macrophages on the bacterial burden, as well as of TMEM175 inhibition on mortality and bacterial burden. Lastly, in vitro experiments with TMEM175‐knockdown macrophages were performed to further elucidate its effects on phagocytosis, lysosomal acidification, cathepsin activity, and bacterial clearance.

## 2. Materials and Methods

### 2.1. Isolation of Human PBMCs

Between October 2023 and October 2024, we enrolled 44 patients with Gram‐negative sepsis at the ICU of Peking University Shenzhen Hospital. All subjects were screened within 12 h of admission and met the Sepsis‐3 diagnostic criteria, presenting with confirmed infection and elevated SOFA scores. Exclusion criteria encompassed age <18 or >80 years, pregnancy or lactation, HIV infection, exposure to corticosteroids or chemotherapy within the previous 30 days, and an inability to grant informed consent. Comprehensive metrics were documented (Table S1). The hospital’s Clinical Research Ethics Committee approved the study [2023‐110‐01], and informed consent was obtained from all participants or their proxies.

Upon ICU admission, whole blood was collected to isolate PBMCs and subsequent monocytes via Ficoll density gradient centrifugation. Finally, TMEM175 mRNA expression was quantified using real‐time qRT‐PCR and normalized against endogenous *β*‐actin. Primer sequences are detailed in Table S2.

### 2.2. Cell Line Culture

iBMDM cells were cultured in high‐glucose DMEM (G4511‐500 ML, Servicebio, China) supplemented with 10% fetal bovine serum (FBS, A5669201, Gibco, USA) and 1% penicillin‐streptomycin (P/S, 100 U/mL and 100 µg/mL, G4003‐100 ML, Servicebio, China). The cells were cultured in a 37°C, 5% CO_2_, saturated humidity incubator and passaged every 1–2 days (cell confluence reached 80%–90%).

Primary bone marrow‐derived macrophages (BMDMs) were isolated from 8‐week‐old C57BL/6J mice (18–22 g, SPF grade) in accordance with the previously described protocol [25]. All animal experimental procedures were approved by the Institutional Animal Care and Use Committee (IACUC) of Shenzhen TopBiotech Co., Ltd. (Ethics Approval No: TOP‐IACUC‐2022‐0241; Experimental Animal Use License Nos: SYXK (Yue) 2020‐0230 (Nanshan)). Briefly, mice were humanely euthanized via carbon dioxide asphyxiation in a chamber with gradual gas filling. Death was confirmed by the absence of a heartbeat, respiratory arrest, and no response to a firm paw pinch for 5 min. Subsequently, the femurs and tibias were aseptically dissected. Bone marrow was gently flushed from the marrow cavities using sterile DMEM, and the resulting marrow cells were collected by centrifugation at 300 × *g* for 3 min. After lysing erythrocytes with hypotonic buffer, cells were resuspended in complete DMEM medium supplemented with 10% FBS, 1% P/S, and 10 ng/mL recombinant mouse macrophage colony‐stimulating factor (M‐CSF, CB34, Novoprotein, China). These cells were then induced to differentiate for 7 days at 37°C in a 5% CO_2_ incubator, with the medium changed every 48 h.

### 2.3. Gene Silencing by siRNA Transfection

Cells were transiently transfected with TMEM175 siRNA or negative control (NC) siRNA using Lipofectamine RNAiMAX Reagent (13778150, Thermo Fisher Scientific, USA) according to the manufacturer’s protocol. The siRNA sequences targeting TMEM175 were designed as follows: 5^′^‐UUGGAUCUGUGCAGGCAAUTT‐3^′^ (sense) and 5^′^‐AUUGCCUGCACAGCAUCCAATT‐3^′^ (antisense). For each transfection, 10 pmol of siRNA was mixed with serum‐free Opti‐MEM medium (31985070, Gibco, China) containing 3 μL transfection reagent. The mixture was incubated at room temperature for 20 min before being transferred to cells seeded in 24‐well plates. After 48 h, the transfection efficiencies were validated by quantitative real‐time polymerase chain reaction (qPCR) analysis. The transfected cells were obtained for subsequent experiments after 72 h of culture.

### 2.4. Lentiviral Transfection of iBMDMs

iBMDM cells were seeded at an appropriate density in cell culture plates and cultured in a complete medium supplemented with 10% FBS at 37°C in 5% CO_2_ until reaching 50%–60% confluency. Lentiviral particles carrying the *TMEM175* gene and a GFP tag (Overexpression lentiviral particles, LV‐TMEM175‐OE, Obio Technology, China) were transduced into the cells at an optimal multiplicity of infection (MOI = 10). Following a 12 h transduction period, the medium was replaced with a fresh complete medium and maintained under standard culture conditions for an additional 48 h. Selection was initiated by adding puromycin (10 μg/mL, ST551, Beyotime, China) to the culture medium, and cells were cultured under continuous drug pressure for 5–7 days. During the selection period, the drug‐containing medium was regularly replaced, and GFP expression was observed under a fluorescence microscope to assess the infection efficiency. Finally, Western Blot analysis was performed to confirm TMEM175 protein overexpression, thereby verifying the successful establishment of the stable cell line.

### 2.5. Gentamicin Protection Assay

si‐NC and si‐TMEM175 BMDMs were seeded in 24‐well plates and incubated with *Escherichia coli* (MOI = 20) for 1 h. Cells were then rinsed with PBS and incubated in medium containing Gentamicin (20 µg/mL, IR9101, Solarbio, China) at 37°C for an additional hour to clear extracellular bacteria. Thereafter, the cells were lysed with 1% Triton X‐100, and intracellular bacteria were enumerated by plating serial dilutions on agar plates. CFUs calculated at 1 h indicated the total uptake of bacteria (phagocytosis). CFUs at 3 and 6 h denoted the survival rate of intracellular bacteria at the respective time points.

### 2.6. Flow Cytometry for Macrophage Purity

To verify the purity of the isolated BMDMs and assess neutrophil contamination, cultured cells were harvested on day 7, washed with PBS, and stained with fluorochrome‐conjugated antibodies against mouse CD11b (101207, BioLegend, USA, 1:200 dilution) and Ly6G (A264, Abmart, China, 1:200 dilution) for 30 min at 4°C in the dark. Cells were analyzed using a flow cytometer to confirm the macrophage population (CD11b^+^/Ly6G^−^) and rule out neutrophil (Ly6G^+^) contamination.

### 2.7. Detection of Mitochondrial Membrane Potential and ROS Levels

Mitochondrial membrane potential (ΔΨm) and intracellular reactive oxygen species (ROS) levels were evaluated using the JC‐1 (C2005, Beyotime, China) and H2DCFDA (HY‐D0940, MedChemExpress, USA) fluorescent probes, respectively, according to the manufacturer’s protocols. Briefly, si‐NC and si‐TMEM175 BMDMs were seeded in 96‐well black‐bottomed plates and incubated with the respective dyes at 37°C in the dark. For ΔΨm assessment, cells were incubated with JC‐1 dye for 20 min; for ROS detection, cells were incubated with H2DCFDA for 30 min. After washing with PBS to remove extracellular dyes, the fluorescence was measured using a microplate reader. The excitation and emission wavelengths were set at 488/530/590 nm for JC‐1 (ratio of 590 to 530 nm) and 492/517 nm for H2DCFDA. Intracellular ROS levels were calculated based on the fluorescence intensity normalized to the total protein content.

### 2.8. Lysosomal pH Detection

BMDMs were seeded to a density of 5 × 10^4^ cells/well in 96‐well black‐bottomed clear plates (3340, Corning, USA) and cultured in a complete medium at 37°C in 5% CO_2_. Following medium removal, 50 μL of 1:1000 diluted Lysosensor Yellow/Blue DND‐160 (L7545, Thermo Fisher Scientific, USA) dye working solution was added to each well. The dye was discarded, 50 μL of fresh culture medium was added, and the plate was immediately detected on a microplate reader (Synergy H, USA). The excitation wavelength was 360 nm, and the emission wavelengths were 451 and 518 nm, respectively. Lysosomal pH was calculated as the ratio of emission intensities at 518 to 451 nm.

## 3. Sepsis Model

### 3.1. Experimental Animals

C57BL/6 mice were obtained from GemPharmatech Co., Ltd. (Guangzhou, China). Animals were housed in SPF facility under a 12 h light/12 h dark cycle at 22 ± 2°C with 50 ± 10% humidity with free access to water and food and acclimated for at least 7 days before use. All animal experiments were approved by the IACUC of Shenzhen TopBiotech Co., Ltd.

### 3.2. CLP

The classic CLP model was established as described previously [26]. Eight‐week‐old C57BL/6J mice (18–22 g, specific pathogen‐free [SPF] grade) were used in this study. All animal procedures were approved by the IACUC of Shenzhen TopBiotech Co., Ltd. (Ethics Approval No: TOP‐IACUC‐2022‐0240; Experimental Animal Use License Nos: SYXK (Yue) 2020‐0230 (Nanshan)) and conducted in compliance with the 3R principles (replacement, reduction, and refinement). Briefly, mice were fasted for 8 h and deprived of water for 4 h prior to surgery. Anesthesia was induced with 4% isoflurane in an induction chamber (1–2 L/min O_2_) and maintained at 2% isoflurane via a nose cone (0.5–1 L/min O_2_) throughout the procedure. Under isoflurane anesthesia, the animal was positioned on the operating table. Following skin disinfection, a 1 cm incision was made along the abdominal midline to expose the cecum. The cecum was ligated midway between its distal pole and base and was subsequently punctured once with a 5 mL syringe needle distal to the ligation site. A small amount of fecal content was extruded through the puncture, after which the cecum was returned to the abdominal cavity. The peritoneal and muscular layers were sutured closed, followed by postoperative skin disinfection. Sham‐operated animals underwent identical procedures excluding ligation and puncture.

### 3.3. Infection With *E. coli*

Six‐to‐eight‐week‐old C57BL/6 mice were challenged with an intraperitoneal injection of 2 × 10^7^ CFUs of *E. coli*. After 6 h, the peritoneal cavity was lavaged with PBS.

### 3.4. Adoptive Transfer of TMEM175‐Overexpressing iBMDM

Macrophage reconstitution was carried out using the method described previously [27]. Briefly, to deplete endogenous macrophages, clodronate liposomes (200 μL/mouse, 40337ES10, Yeasen, China) were administered intraperitoneally to WT mice for macrophage depletion. Two days post‐depletion, 5 × 10^6^ empty‐vector‐transduced or TMEM175‐overexpressing iBMDMs were intraperitoneally injected into macrophage‐depleted WT mice, followed by CLP induction 6 h later to establish the sepsis model.

### 3.5. Pharmacological Inhibition of TMEM175 With 2‐phenylcyclopentylamine (2‐PPA) In Vivo

Mice were intraperitoneally injected with the TMEM175‐specific inhibitor 2‐PPA [28] 12 h before CLP (HY‐33878, MedChemExpress, USA). For the assessment of 7‐day survival, an additional dose of 2‐PPA was administered at 24 h post‐CLP. 2‐PPA was prepared in PBS containing 1% DMSO at a dose of 5 mg/kg. Control mice were injected with an equal volume of the solvent. No significant behavioral abnormalities were observed in any mice post‐administration. At 24 h post‐treatment, organ bacterial loads were quantified.

Seven‐day survival rates were recorded for mice. Post‐surgery, the survival status of mice was monitored continuously for 7 days: their health condition and behavioral changes were observed every 6 h within the first 72 h after CLP and every 12 h thereafter. The primary humane endpoints were defined in accordance with the approved animal welfare protocol, including severe and persistent lethargy with no response to external stimuli, rapid weight loss (15%–20% of the original body weight), lying prostrate without movement, convulsions, paralysis, dyspnea, or core body temperature <32°C; mice meeting any of these humane endpoints were immediately euthanized via carbon dioxide asphyxiation to minimize suffering, in line with the 3R principles and ethical requirements.

### 3.6. Peritoneal Lavage Fluid (PLF)

After the mouse was killed, it was fixed on a dissection board, and the abdominal skin was disinfected with 75% alcohol. The skin and peritoneum were opened with sterile surgical scissors to expose the abdominal cavity. Slowly inject 5 mL of 4°C pre‐chilled sterile PBS into the peritoneal cavity. Gently massage the abdomen for 1 min to ensure thorough contact of the fluid with the peritoneal contents. Then, carefully aspirate the PLF with a syringe and transfer it to a 15 mL centrifuge tube. Repeat this procedure three times, ensuring an 80% recovery rate each time. The resulting lavage fluid was centrifuged at 300 × *g* for 3 min, and the cell pellet was used for subsequent experiments.

### 3.7. Bacterial Burden Assay

Following humane euthanasia via carbon dioxide asphyxiation under deep isoflurane anesthesia, whole blood (0.8–1.0 mL per mouse) was collected from mice (16–18 g at sacrifice, 24 h after CLP). To obtain the PLF, 10 mL of sterile 0.1 M PBS was gently instilled into the peritoneal cavity via catheterization, followed by aspiration to collect the PLF. The liver, spleen, and lung were dissected, rinsed briefly with ice‐cold sterile PBS to remove surface contaminants, and immediately weighed to determine the wet weight. Harvested tissues were homogenized in 5 mL of PBS per 0.5 g of wet tissue using a homogenizer (Bona Tech S10, China). Serial tenfold dilutions (10^1^ to 10^2^) were prepared in sterile PBS for each sample. 50 µL from each serial dilution was dispensed onto LB agar plates using a micropipette. After incubation at 37°C in a 5% CO_2_ incubator overnight, CFUs were counted.

### 3.8. Western Blot

After rinsing the cells with PBS, the cells were lysed for 30 min using RIPA lysis buffer containing 1 mM PMSF on ice. The lysates were centrifuged at 12,000 × *g* and 4°C for 10 min to collect the supernatant for protein quantification. Protein was separated via SDS‐PAGE and transferred onto PVDF (IPFL00005, Millipore, USA) membranes. The membranes were blocked with 5% BSA (GC305010, Servicebio, China) for 2 h at room temperature, after which they were incubated with primary antibodies overnight at 4°C. Following washing with TBST (G0004, Servicebio, China), the membranes were incubated with HRP‐conjugated secondary antibodies (anti‐rabbit:GB23303; anti‐mouse:GB23301, Servicebio, China) for 2 h at room temperature. Protein bands were visualized using ECL chemiluminescence reagents (G2074, Servicebio, China), and grayscale analysis was conducted using ImageJ software. The primary antibodies utilized in this study are listed in Table S3.

### 3.9. qPCR

Total RNA was isolated using the NcmSpin Rapid RNA Kit (M5105, NCM Biotech, China), and its concentration and purity (A260/A280 ratio) were determined on a NanoDrop 2000 spectrophotometer (Thermo Fisher Scientific, Waltham, MA, USA). Subsequently, 1 µg of RNA was reverse‐transcribed into first‐strand cDNA using the SPARKscript Ⅱ RT Plus Kit (AG0304‐C, Sparkjade, China) according to the manufacturer’s instructions. qPCR was carried out with the SYBR Green qPCR Master Mix (G3326‐15, Servicebio, China) on a CFX96 real‐time system (Bio‐Rad). *β*‐Actin was used as the internal reference gene for normalization in the 2^−ΔΔCt^ calculation of relative mRNA abundance. All primer sequences are listed in Table S2.

### 3.10. RNA Sequencing

BMDMs were seeded in six‐well plates (80%–90% confluency) and transfected with TMEM175 siRNA or NC siRNA. At 72 h post‐transfection, cells were either left uninfected or infected with *E. coli* (MOI = 20) for 6 h, thereby creating four experimental groups (*n* = 4). Total RNA was isolated via TRIzol, qualified by NanoDrop 2000 (A260/A280 1.8–2.0, A260/A230 >2.0) and Agilent 2100 (RIN ≥8.0). cDNA libraries (5 μg RNA/sample) were built by enriching mRNA, fragmenting, synthesizing cDNA, adding adapters, and PCR amplifying and then sequenced on Illumina NovaSeq 6000 (PE150, 6 Gb/sample). Raw reads were filtered (Trimmomatic: remove adapters, *Q* ≤ 20 bases, <50 bp reads), aligned to GRCm38 (HISAT2), and assembled (StringTie). Functional annotation was performed using DIAMOND (NR/Swiss‐Prot), hmmscan (Pfam), and InterProScan (GO‐term assignment). Differential expression (DESeq2/edgeR/EBSeq) defined DEGs as |log_2_FC|>1, adj. *p* < 0.05. Enrichment (clusterProfiler) and all analyses were done in R (supported by GO/KEGG/STRING).

### 3.11. Statistical Analysis

Single‐cell RNA‐seq data of GSE217906 were analyzed in R (Seurat, Harmony). Raw data were organized, merged into Seurat objects, and filtered (nFeature_RNA: 200–8000; mt% <15%). After normalization, HVG selection, and harmony batch correction, PCA/UMAP dimensionality reduction, and graph‐based clustering (resolution = 0.5) were performed. Clusters were annotated into immune subtypes via marker genes. TMEM175 expression was visualized and compared between healthy control (HC) and sepsis groups. To further define monocyte subtypes, cells annotated as monocytes were extracted and subjected to sub‐clustering. After re‐normalization, variable feature selection, and PCA, UMAP was re‐computed using the first 15 principal components. Graph‐based clustering (resolution = 0.3) was performed on the monocyte subset, yielding 15 sub‐clusters. These sub‐clusters were manually annotated into classical monocytes (FCN1‐high/VCAN‐high/S100A8‐high/FCGR3A‐low), intermediate monocytes (FCGR3A‐moderate/HLA‐DRA‐high), and nonclassical monocytes (FCGR3A‐high/FCN1‐low) based on canonical marker expression. The proportion of each monocyte subtype was calculated per patient to assess inter‐individual compositional variation. TMEM175 expression levels were compared across monocyte subtypes between the HC and sepsis groups.

Numerical data were expressed as the mean ± standard deviation (SD). All statistical analyses were performed using GraphPad Prism software (version 9.5.0, USA). Comparisons between two groups were made using unpaired, two‐tailed Student’s *t* tests, while comparisons among multiple groups or under multiple conditions involving two independent variables were conducted using one‐way or two‐way analysis of variance (ANOVA), followed by Šídák’s post hoc test for multiple comparisons. Mouse survival curves were compared using the log‐rank (Mantel–Cox) test. A *p*‐value of less than 0.05 was considered statistically significant.

## 4. Results

### 4.1. The Expression of TMEM175 Is Downregulated in Septic Patients

**Figure 1:**
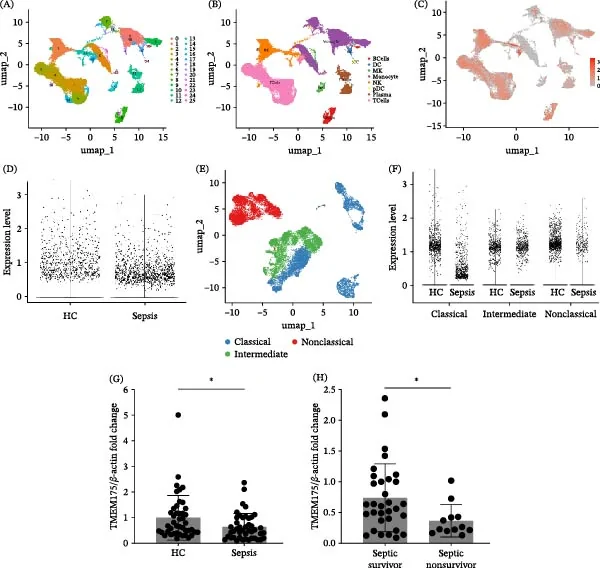
TMEM175 expression is downregulated in septic patients. (A) Single‐cell RNA sequencing data from dataset GSE217906 were visualized using UMAP, showing 25 distinct clusters of peripheral blood mononuclear cells (PBMCs). (B) Cell clusters were annotated into major immune cell types: B cells, DC, MK, Monocytes, NK, pDC, Plasma, and T cells. (C) TMEM175 expression distribution across PBMC subsets was visualized on UMAP coordinates, with red indicating high expression levels. (D) TMEM175 expression in monocytes from healthy controls (HC) and sepsis was compared using a dot plot. (E) UMAP visualization of monocytes after sub‐clustering, identifying three subsets: classical monocytes, intermediate monocytes, and nonclassical monocytes. (F) Violin plot of TMEM175 expression across classical, intermediate, and nonclassical monocyte subsets in HC and sepsis. (G) TMEM175 mRNA levels in PBMCs from HC (*n* = 44 biologically independent donors) and sepsis (*n* = 44 biologically independent donors) were assessed by quantitative real‐time polymerase chain reaction. Statistical significance was determined by unpaired *t* test. Error bars represent mean ± SD. (H) TMEM175 mRNA levels in PBMCs from septic survivors (*n* = 32 biologically independent donors) and septic nonsurvivors (*n* = 12 biologically independent donors) were assessed by quantitative real‐time polymerase chain reaction. Statistical significance was determined by unpaired *t*‐test. Error bars represent mean ± SD.  ^∗^ *p* < 0.05. The exact *p*‐value for panel G (HC vs. sepsis) is 0.0211. The exact *p*‐value for panel H (septic survivor vs. septic nonsurvivor) is 0.0309.

To investigate the role of TMEM175 in sepsis progression, we first analyzed scRNA‐seq data from five septic patients and five HCs, which was obtained from the public dataset GSE217906 (GEO database). By integrating immune marker annotations, we classified PBMCs into eight functional subpopulations (Figure 1A,B). TMEM175 was highly expressed in B cells, T cells, dendritic cells, natural killer cells, and monocytes (Figure 1C). Given monocytes’ critical role in sepsis pathogenesis, we further examined TMEM175 expression and found that it trended toward lower levels in monocytes from patients with sepsis than in those from HC (Figure 1D, *p* = 0.058). To determine which monocyte subset primarily accounts for this reduction, we sub‐clustered monocytes and identified three transcriptionally distinct subsets—classical, intermediate monocytes, and nonclassical monocytes (Figure 1E). Notably, TMEM175 downregulation was predominantly observed in classical monocytes (Figure 1F), which were also proportionally expanded in septic patients relative to HC, whereas the nonclassical subset was correspondingly reduced (Figure S1). To confirm these findings in clinical samples, we measured TMEM175 mRNA expression in PBMCs isolated from healthy volunteers and Gram‐negative septic patients. Consistent with the scRNA‐seq data, TMEM175 mRNA expression was significantly downregulated in septic patients’ PBMCs relative to HC (Figure 1G). Building on the finding that TMEM175 is downregulated in sepsis, we further observed that survivors of sepsis exhibited higher levels of TMEM175 mRNA expression compared to nonsurvivors (Figure 1H).

### 4.2. TMEM175‐Expression in Macrophages Protects Mice Against Sepsis

**Figure 2:**
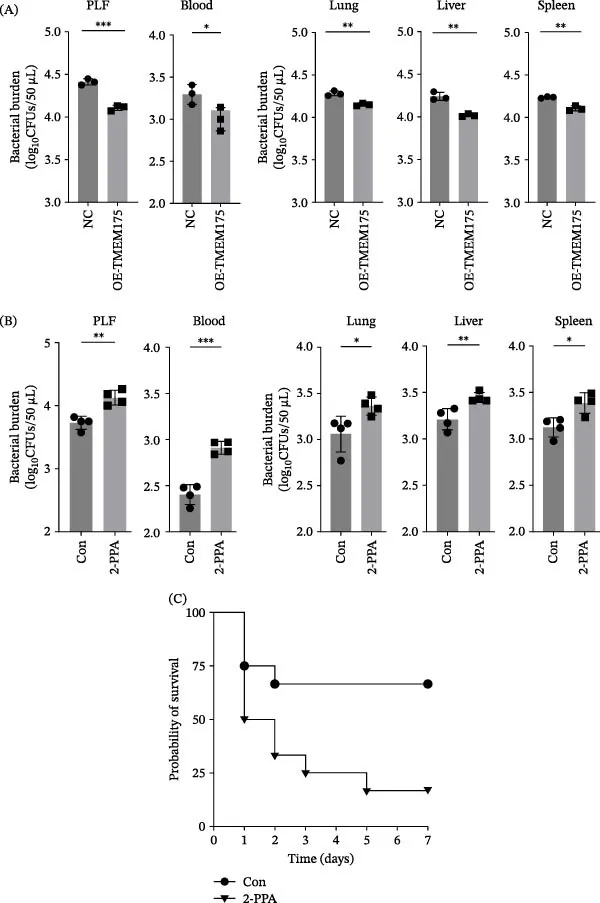
TMEM175‐expression in macrophages protects mice against sepsis. (A) Mice were pretreated with a macrophage‐depleting agent 48 h pre‐infection, followed by intraperitoneal TMEM175‐overexpressing/negative control (NC) iBMDMs 6 h before cecal ligation and puncture (CLP). Bacterial burdens in PLF, lung, liver, and spleen were assessed 24 h post‐CLP (*n* = 3 biologically independent mice per group). Statistical significance was determined by two‐way ANOVA with Šídák’s multiple comparisons post hoc test. Error bars represent mean ± SD. (B) Mice received an intraperitoneal injection of 2‐PPA 12 h before CLP surgery. Bacterial burdens in the PLF, lung, liver, spleen, and blood were determined 24 h post‐infection (*n* = 4 biologically independent mice per group). Statistical significance was determined by two‐way ANOVA with Šídák’s multiple comparisons post hoc test. Error bars represent mean ± SD. (C) Mice received an intraperitoneal injection of 2‐PPA 12 h before CLP surgery (*n* = 6 biologically independent mice per group). Survival curves of each group are presented in Kaplan–Meier plots with log‐rank (Mantel–Cox) test.  ^∗^ *p* < 0.05,  ^∗∗^ *p* < 0.01,  ^∗∗∗^ *p* < 0.001. The exact *p*‐values for panel A (NC vs. OE‐TMEM175) are: PLF (*p* = 0.0004), blood (*p* = 0.0497), lung (*p* = 0.0020), liver (*p* = 0.0017), and spleen (*p* = 0.0015). The exact *p*‐values for panel B (Con vs. 2‐PPA) are: PLF (*p* = 0.0020), blood (*p* = 0.0002), lung (*p* = 0.0315), liver (*p* = 0.0091), and spleen (*p* = 0.0133). The exact *p*‐value for panel C is 0.0196.

To evaluate TMEM175’s role in sepsis in vivo, we depleted macrophages in mice and performed adoptive transfer of TMEM175‐overexpressing iBMDMs. Compared with mice receiving empty vector control cells, TMEM175‐overexpressing iBMDMs significantly reduced the bacterial load in PLF, lung, liver, spleen, and blood of septic mice (Figure 2A).

Conversely, a systemically increased bacterial burden was observed following the pharmacological blockade of TMEM175 by 2‐PPA on day 1 (Figure 2B). Consistent with these results, we also observed a significant reduction in the survival rate. 2‐PPA‐treated mice showed a higher mortality of 83% within 7 days post‐CLP, compared to only 33% in the WT mice (Figure 2C).

### 4.3. TMEM175 Expression Is Downregulated in Macrophages After Experimental Sepsis

**Figure 3:**
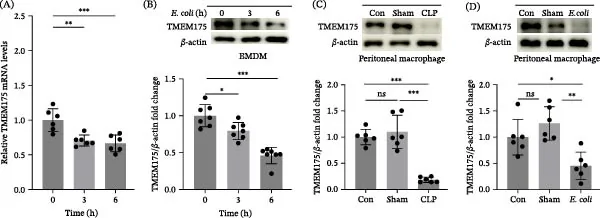
TMEM175 expression is dynamically downregulated after in vitro and in vivo sepsis in macrophages. (A, B) BMDMs were stimulated with live *E. coli* (MOI = 20) for 3 and 6 h. (A) TMEM175 mRNA levels were assessed by quantitative real‐time polymerase chain reaction (*n* = 6 biologically independent samples per group). Statistical significance was determined by one‐way ANOVA with Šídák’s multiple comparisons post hoc test. Error bars represent mean ± SD. (B) TMEM175 protein levels were assessed by Western Blot and quantified relative to *β*‐actin (*n* = 7 biologically independent samples per group). Statistical significance was determined by one‐way ANOVA with Šídák’s multiple comparisons post hoc test. Error bars represent mean ± SD. (C) Mice were subjected to sham surgery or CLP. PLF was collected 24 h later. TMEM175 protein levels were assessed by Western Blot and quantified relative to *β*‐actin (*n* = 6 biologically independent samples per group). Statistical significance was determined by unpaired *t*‐test. Error bars represent mean ± SD. (D) PLF was collected 6 h after intraperitoneal injection of live *E. coli* (2 × 10^7^ CFU per mouse). TMEM175 protein levels were assessed by Western Blot and quantified relative to *β*‐actin (*n* = 6 biologically independent samples per group). Statistical significance was determined by unpaired *t* test. Error bars represent mean ± SD.  ^∗^ *p* < 0.05,  ^∗∗^ *p* < 0.01,  ^∗∗∗^ *p* < 0.001. The exact *p*‐values for panel A are: 0 vs. 3 h (*p* = 0.0019), and 0 vs. 6 h (*p* = 0.0006). The exact *p*‐values for panel B are: 0 vs. 3 h (*p* = 0.0131), and 0 vs. 6 h (*p* = 0.0002). The exact *p*‐values for panel C are: Con vs. Sham (*p* = 0.8026), Sham vs. CLP (*p* = 0.0002), and Con vs. CLP (*p* = 0.0003). The exact *p*‐values for panel D are: Con vs. Sham (*p* = 0.4139), Sham vs. *E. coli* (*p* = 0.0012), and Con vs. *E. coli* (*p* = 0.0226).

Inflammation triggers monocytes to differentiate into macrophages, which function as critical sentinel cells in the innate immune system to combat infections. In BMDMs, TMEM175 mRNA and protein levels were significantly downregulated at both 3 h time points post‐live *E. coli* challenge (Figure 3A,B). Additionally, peritoneal macrophages from CLP‐induced polymicrobial septic mice showed markedly reduced TMEM175 expression after sepsis onset (Figure 3C). Furthermore, TMEM175 expression was significantly downregulated in peritoneal macrophages during *E. coli*‐induced peritonitis (Figure 3D). These results demonstrate that TMEM175 expression in macrophages is dynamically regulated by infection and inflammation, with pronounced downregulation across multiple sepsis models.

### 4.4. TMEM175‐Knockdown Impairs Macrophage Bactericidal Activity and Organelle Homeostasis

To confirm that the observed phenotypes were macrophage‐specific, flow cytometry was utilized to quantify neutrophil contamination. Our cultured BMDM preparation demonstrated high purity, consisting of 96.9% CD11b^+^/Ly6G^−^ macrophages with minimal (0.1%) Ly6G^+^ neutrophil contamination. (Figure S2). We next assessed the phagocytic and bactericidal functions of si‐TMEM175 BMDMs. Following phagocytosis of live *E. coli*, the mean CFUs in si‐TMEM175 and si‐NC macrophages were comparable (Figure 4A). However, si‐TMEM175 macrophages harbored significantly more intracellular bacteria after infection with live *E. coli* for 6 h (Figure 4B). These results indicate that *TMEM175* gene knockdown impairs macrophage bactericidal activity. CTSD, a broad‐spectrum antimicrobial protein critical for macrophage bactericidal activity [29], was examined for its regulation by TMEM175. si‐TMEM175 macrophages exhibited reduced mature CTSD levels following *E. coli* phagocytosis compared to si‐NC (Figure 4C), suggesting that impaired CTSD maturation contributed to the defective bactericidal capacity in TMEM175‐deficient macrophages. To determine whether TMEM175 regulates lysosome quantity, we assessed LAMP‐1 expression and found no significant difference between si‐TMEM175 and si‐NC macrophages (Figure 4D), suggesting that the bactericidal defect arises from impaired lysosomal function rather than reduced lysosome abundance. Since terminal autophagic degradation strictly relies on functional lysosomal hydrolases like CTSD [30], we suspected that impaired CTSD maturation might trigger a secondary blockade in the autophagic flux. Indeed, si‐TMEM175 macrophages exhibited an elevated LC3B‐II/I ratio and marked accumulation of p62, a difference that was abolished following Baf A1 treatment (Figure 4F), thereby confirming a severe impairment in autophagic degradation. This failure in autophagic clearance inevitably compromises the removal of damaged organelles [31]. Supporting this, JC‐1 staining showed a significant drop in mitochondrial membrane potential following si‐TMEM175 (Figure 4E), accompanied by a substantial elevation in intracellular ROS levels (Figure 4G). We initially hypothesized that this excessive ROS might act as a toxic mediator, exacerbating the bactericidal defect. However, neutralizing ROS with the antioxidant NAC failed to rescue mature CTSD levels and paradoxically worsened intracellular bacterial survival (Figure 4H,I). Together, these results suggest that the elevated ROS are not a pathogenic driver of the bactericidal defect but rather an essential—though insufficient—compensatory antimicrobial response [32] deployed when lysosomal proteolysis is compromised.

Figure 4TMEM175‐knockdown impairs macrophage bactericidal activity and organelle homeostasis. BMDMs were transfected with negative control siRNA (si‐NC) or TMEM175‐targeting siRNA (si‐TMEM175) (A, B) si‐NC and si‐TMEM175 BMDMs were infected with live *E. coli* (MOI = 20), and bacterial survival was assessed by CFUs assay (*n* = 4 biologically independent samples per group). Statistical significance was determined by two‐way ANOVA with Šídák’s multiple comparisons post hoc test. Error bars represent mean ± SD. (C, D) si‐NC and si‐TMEM175 BMDMs were infected with live *E. coli* (MOI = 20). Mature CTSD levels (C) were analyzed by Western Blot and quantified relative to *β*‐actin (*n* = 4 biologically independent samples per group). LAMP‐1 expression (D) was assessed by Western Blot and quantified relative to *β*‐actin (*n* = 4 biologically independent samples per group). Statistical significance was determined by unpaired *t*‐ test. Error bars represent mean ± SD. (E) si‐NC and si‐TMEM175 BMDMs were infected with live *E. coli* (MOI = 20). Mitochondrial membrane potential in si‐NC and si‐TMEM175 BMDMs was assessed by JC‐1 staining (*n* = 12 biologically independent samples per group). Statistical significance was determined by two‐way ANOVA with Šídák’s multiple comparisons post hoc test. Error bars represent mean ± SD. (F) si‐NC and si‐TMEM175 BMDMs were pre‐treated with or without Bafilomycin A1 (100 nM) and infected with live *E. coli* (MOI = 20). p62 and LC3B levels were analyzed by Western Blot, and the fold changes of p62/*β*‐actin and LC3B‐II/LC3B‐I were quantified (*n* = 3 biologically independent samples per group). Statistical significance was determined by two‐way ANOVA with multiple comparisons post hoc test. Error bars represent mean ± SD. (G) si‐NC and si‐TMEM175 BMDMs were infected with live *E. coli* (MOI = 20). Relative ROS levels were measured at the indicated times (*n* = 12 biologically independent samples per group). Statistical significance was determined by two‐way ANOVA with Šídák’s multiple comparisons post hoc test. Error bars represent mean ± SD. (H) si‐NC and si‐TMEM175 BMDMs were pre‐treated with or without N‐acetylcysteine (10 mM) and infected with live *E. coli* (MOI = 20). Bacterial survival was assessed by CFUs assay (*n* = 4 biologically independent samples per group). Statistical significance was determined by two‐way ANOVA with multiple comparisons post hoc test. Error bars represent mean ± SD. (I) si‐NC and si‐TMEM175 BMDMs were pre‐treated with or without NAC and infected with live *E. coli* (MOI = 20). Mature CTSD levels were analyzed by Western Blot and quantified relative to *β*‐actin (*n* = 3 biologically independent samples per group). Statistical significance was determined by two‐way ANOVA with multiple comparisons post hoc test. Error bars represent mean ± SD.  ^∗^ *p* < 0.05, *p* < 0.01,  ^∗∗∗^ *p* < 0.001,  ^∗∗∗∗^ *p* < 0.0001. The exact *p*‐values for panel A (si‐NC vs. si‐TMEM175) are: 0.5 h (*p* = 0.9780), 1 h (*p* = 0.4711), and 3 h (*p* = 0.9552). The exact *p*‐values for panel B (si‐NC vs. si‐TMEM175) are: 0 h (*p* = 0.8251), 3 h (*p* = 0.5925), and 6 h (*p* = 0.0043). The exact *p*‐values for panel C (si‐NC vs. si‐TMEM175) are: 0 min (*p* = 0.000837), 30 min (*p* = 0.020158), 60 min (*p* = 0.034670), and 90 min (*p* = 0.04789). The exact *p*‐values for panel D (si‐NC vs. si‐TMEM175) are: 0 min (*p* = 0.585773), 30 min (*p* = 0.654053), 60 min (*p* = 0.742183), and 90 min (*p* = 0.654053). The exact *p*‐values for panel E (si‐NC vs. si‐TMEM175) are: 0 h (*p* = 0.0005), and 2 h (*p* < 0.0001). The exact *p*‐values for panel F (p62/*β*‐actin) are: 0 h (si‐NC vs. si‐TMEM175, *p* = 0.1732; si‐NC + Baf A1 vs. si‐TMEM175+Baf A1, *p* = 0.9891), 2 h (si‐NC vs. si‐TMEM175, *p* = 0.045; si‐NC + Baf A1 vs. si‐TMEM175+Baf A1, *p* = 0.9989), and 4 h (si‐NC vs. si‐TMEM175, *p* = 0.0051; si‐NC + Baf A1 vs. si‐TMEM175+Baf A1, *p* = 0.9998). The exact *p*‐values for panel F (LC3B‐II/LC3B‐I) are: 0 h (si‐NC vs. si‐TMEM175, *p* = 0.0063; si‐NC + Baf A1 vs. si‐TMEM175+Baf A1, *p* > 0.999), 2 h (si‐NC vs. si‐TMEM175, *p* = 0.044; si‐NC + Baf A1 vs. si‐TMEM175+Baf A1, *p* = 0.9166), and 4 h (si‐NC vs. si‐TMEM175, *p* = 0.0004; si‐NC + Baf A1 vs. si‐TMEM175+Baf A1, *p* = 0.9268). The exact *p*‐values for panel G (si‐NC vs. si‐TMEM175) are: 0 min (*p* < 0.0001), 30 min (*p* < 0.0001), and 90 min (*p* < 0.0001). The exact *p*‐values for panel H (at 6 h) are: si‐NC vs. si‐TMEM175 (*p* = 0.0053), and si‐NC + NAC vs. si‐TMEM175+NAC (*p* = 0.0014). The exact *p*‐values for panel I (at 90 min) are: si‐NC vs. si‐TMEM175 (*p* = 0.9759), and si‐NC + NAC vs. si‐TMEM175+NAC (*p* = 0.8504).

**Figure:**
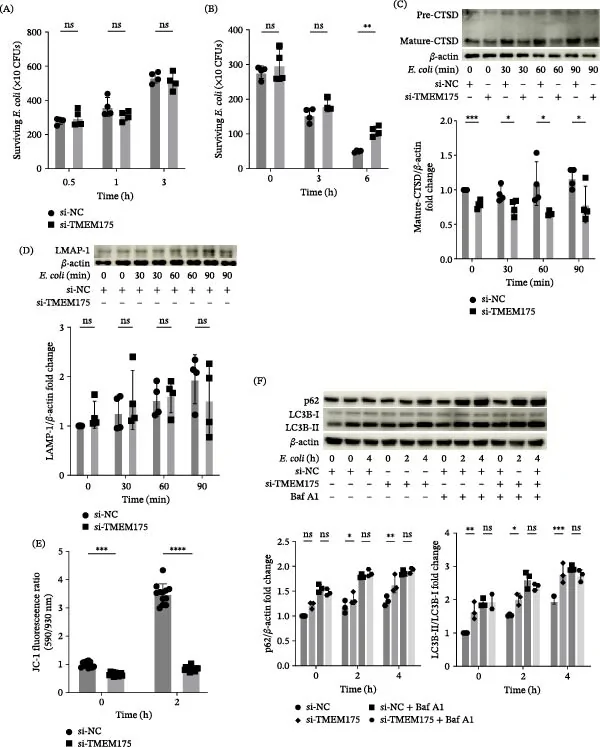

**Figure:**
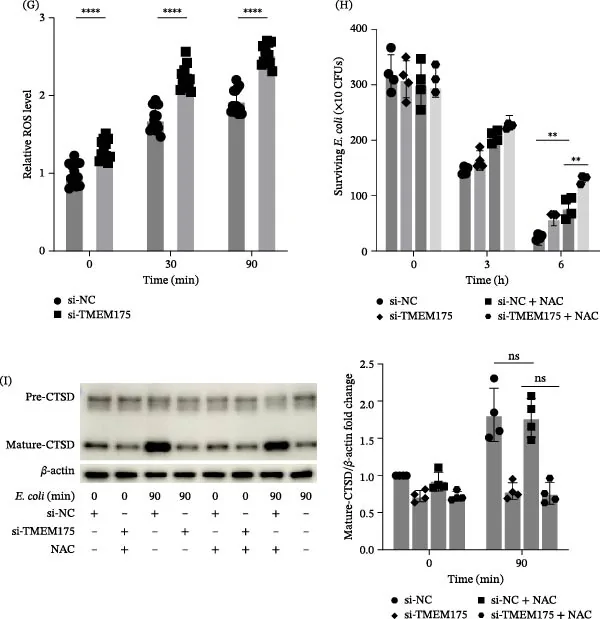

### 4.5. TMEM175 Mediates Transcriptional Regulation of Cathepsins

Following the observed effect of TMEM175 on mature CTSD levels, we investigated the mechanisms underlying TMEM175 knockdown’s impact on macrophage innate immunity using RNA‐seq. RNA‐seq analysis revealed 287 upregulated and 1016 downregulated genes in si‐TMEM175 versus si‐NC BMDMs (fold change ≥2 and FDR <0.01) (Figure 5A,B). KEGG enrichment analysis confirmed that si‐TMEM175 critically regulates endocytosis and phagosome pathways (Figure 5C). Transcriptome analysis revealed significantly reduced expression of multiple cathepsin family members, including CTSD, CTSE, CTSF, CTSS, and CTSH (Figure 5D). This downregulation was validated at the mRNA level in BMDMs and iBMDMs, consistent with the RNA‐seq data (Figure 5E,F). We next assessed the impact of si‐TMEM175 on total CTSD protein levels and lysosomal pH in BMDMs. We found that si‐TMEM175 resulted in both reduced total CTSD protein levels and decreased lysosomal pH (Figure 5G,H). This phenotype is consistent with the findings previously reported in COS‐1 cells [20].

Figure 5TMEM175 mediates transcriptional regulation of cathepsins. (A) RNA‐seq analysis was performed on si‐NC and si‐TMEM175 BMDMs. Volcano plot shows differentially expressed genes, with red dots representing 287 upregulated genes and blue dots representing 1016 downregulated genes (Fold Change ≥ 2 and FDR < 0.01). (B) Heatmap of differentially expressed genes in si‐NC (si‐NC‐1, si‐NC‐2, si‐NC‐3) and si‐TMEM175 (si‐TMEM175‐1, si‐TMEM175‐2, and si‐TMEM175‐3) BMDMs. (C) KEGG pathway enrichment analysis of differentially expressed genes. (D) Expression heatmap of selected cathepsin family members (CTSE, CTSF, CTSH, CTSS, and CTSD) in si‐NC and si‐TMEM175 BMDMs, with blue indicating downregulation. (E, F) Cells were transfected with si‐NC or si‐TMEM175 siRNA. CTS family mRNA levels were detected by quantitative real‐time polymerase chain reaction in BMDMs (E) and iBMDMs (F) (*n* = 6 biologically independent samples per group). (E, F) Statistical significance was determined by two‐way ANOVA with Šídák’s multiple comparisons post hoc test. Error bars represent mean ± SD. (G) Total CTSD protein levels were assessed by Western Blot and quantified relative to *β*‐actin (*n* = 4 biologically independent samples per group). Statistical significance was determined by unpaired *t* test. Error bars represent mean ± SD. (H) A fluorescence‐based assay was performed to measure the pH of lysosome, with the yellow/blue fluorescence ratio (detected by a microplate reader) positively correlating with pH (*n* = 12 biologically independent samples per group). Statistical significance was determined by unpaired *t* test. Error bars represent mean ± SD.  ^∗∗^ *p* < 0.01,  ^∗∗∗^ *p* < 0.001. The exact *p*‐values for panel E (si‐NC vs. si‐TMEM175 in BMDM) are: TMEM175 (*p* = 0.0021), CTSD (*p* = 0.0035), CTSE (*p* = 0.0042), CTSH (*p* = 0.0038), and CTSS (*p* = 0.0047). The exact *p*‐values for panel F (si‐NC vs. si‐TMEM175 in iBMDM) are: TMEM175 (*p* = 0.0018), CTSD (*p* = 0.0029), CTSE (*p* = 0.0033), CTSH (*p* = 0.0040), and CTSS (*p* = 0.0045). The exact *p*‐value for panel G (si‐NC vs. si‐TMEM175) is 0.0003. The exact *p*‐value for panel H (si‐NC vs. si‐TMEM175) is < 0.0001.

**Figure:**
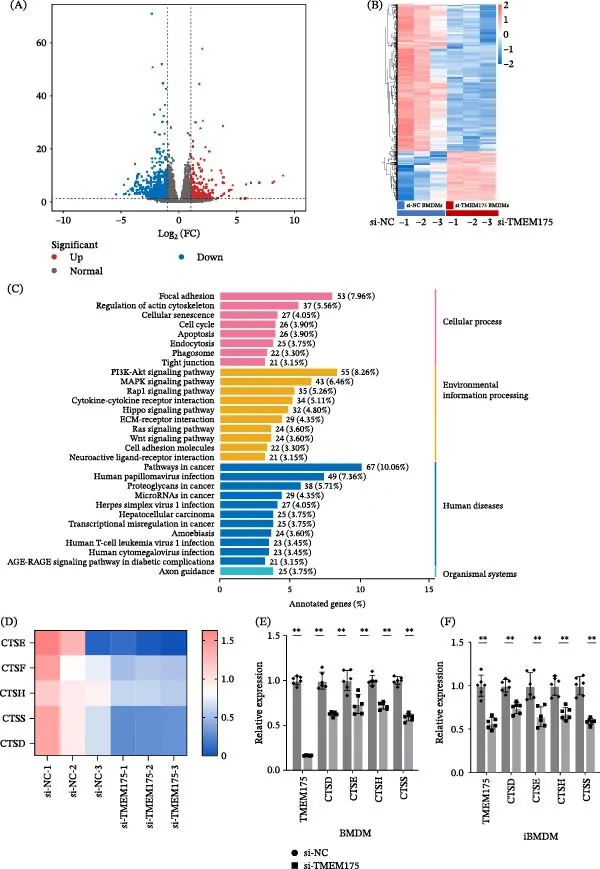

**Figure:**
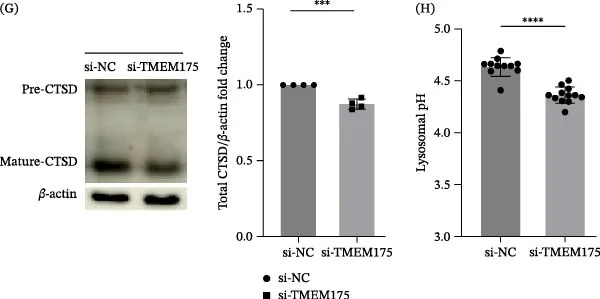

Collectively, these results establish that TMEM175 deficiency in macrophages impairs a critical pathway essential for bacterial killing by mediating the transcriptional downregulation of key cathepsins rather than mere through lysosomal hyper‐acidification.

### 4.6. TMEM175 Deficiency in Macrophages Modulates the Production of Pro‐Inflammatory Cytokines

**Figure 6:**
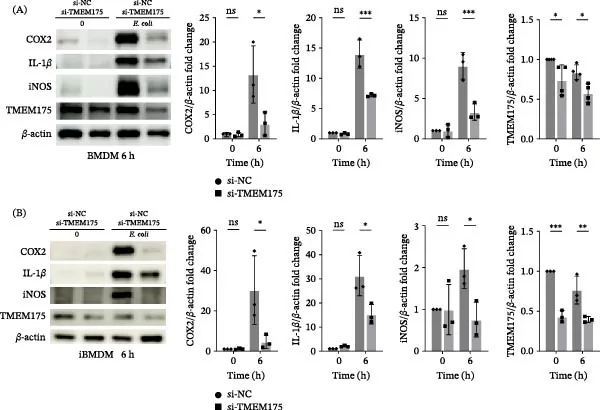
TMEM175 deficiency in macrophages suppresses the production of pro‐inflammatory cytokines upon *E. coli* stimulation. (A) BMDMs were transfected with control si‐NC or si‐TMEM175 siRNA and stimulated with live *E. coli* for 0 or 6 h. Protein levels of COX2, IL‐1*β*, iNOS, and TMEM175 were analyzed by Western Blot and quantified relative to *β*‐actin (*n* = 3 biologically independent samples per group). Statistical significance was determined by two‐way ANOVA with Šídák’s multiple comparisons post hoc test. Error bars represent mean ± SD. (B) iBMDMs were transfected with si‐NC or si‐TMEM175 and stimulated with live *E. coli* for 0 or 6 h. Protein levels of COX2, IL‐1*β*, iNOS, and TMEM175 were analyzed by Western Blot and quantified relative to *β*‐actin (*n* = 3 biologically independent samples per group). Statistical significance was determined by two‐way ANOVA with Šídák’s multiple comparisons post hoc test. Error bars represent mean ± SD.  ^∗^ *p* < 0.05,  ^∗∗^ *p* < 0.01,  ^∗∗∗^ *p* < 0.001. The exact *p*‐values for panel A (si‐NC vs. si‐TMEM175 in BMDM) are: COX2 at 0 h (*p* > 0.9999) and 6 h (*p* = 0.0190); IL‐1*β* at 0 h (*p* = 0.9982) and 6 h (*p* = 0.0004); iNOS at 0 h (*p* > 0.9999) and 6 h (*p* = 0.0007); TMEM175 at 0 h (*p* = 0.0433) and 6 h (*p* = 0.0412). The exact *p*‐values for panel B (si‐NC vs. si‐TMEM175 in iBMDM) are: COX2 at 0 h (*p* > 0.9999) and 6 h (*p* = 0.0280); IL‐1*β* at 0 h (*p* = 0.9923) and 6 h (*p* = 0.0122); iNOS at 0 h (*p* > 0.9999) and 6 h (*p* = 0.0366); TMEM175 at 0 h (*p* = 0.0004) and 6 h (*p* = 0.0066).

Beyond its antibacterial role, TMEM175 activates innate immune responses, as suggested by GO analysis. To investigate its role in sepsis‐related macrophage immunity, we knocked down TMEM175 in BMDMs and stimulated them with live *E. coli*. At 6 h post‐stimulation, Western Blot analysis revealed a marked reduction in protein levels of inflammatory cytokines COX2, IL‐1*β*, and iNOS in si‐TMEM175 BMDMs compared to si‐NC (Figure 6A). Additionally, si‐TMEM175 in iBMDMs resulted in lower COX2, IL‐1*β*, and iNOS levels post‐*E. coli* stimulation (Figure 6B). These findings collectively demonstrate that TMEM175 depletion suppresses the macrophage immune response to *E. coli* infection.

### 4.7. TMEM175 Regulates PI3K‐Akt Signaling Pathway

**Figure 7:**
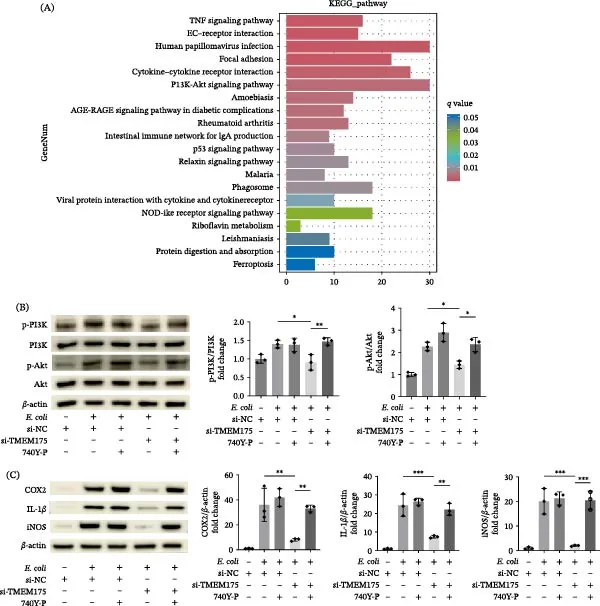
TMEM175‐knockdown dampens pro‐inflammatory cytokine production by impairing PI3K‐Akt signaling pathway activation. (A) KEGG pathway enrichment analysis shows significant enrichment of genes involved in the PI3K‐Akt signaling pathway (B) BMDMs were transfected with control si‐NC or si‐TMEM175 siRNA, pretreated with 740Y‐P (a PI3K agonist) where indicated, and then stimulated with live *E. coli* for 6 h. The phosphorylation levels of PI3K (p‐PI3K) and Akt (p‐Akt) as well as their total protein levels (PI3K, Akt) were assessed by Western Blot and quantified relative to *β*‐actin (*n* = 3 biologically independent samples per group). Statistical significance was determined by ordinary one‐way ANOVA with Tukey’s multiple comparisons test. Error bars represent mean ± SD. (C) BMDMs were transfected with control si‐NC or si‐TMEM175 siRNA, pretreated with 740Y‐P where indicated, and then stimulated with live *E. coli* for 6 h. The protein levels of COX2, IL‐1β, and iNOS were assessed by Western Blot and quantified relative to *β*‐actin (*n* = 3 biologically independent samples per group). Statistical significance was determined by ordinary one‐way ANOVA with Tukey’s multiple comparisons test. Error bars represent mean ± SD.  ^∗^ *p* < 0.05,  ^∗∗^ *p* < 0.01,  ^∗∗∗^ *p* < 0.001. The exact *p*‐values for panel B are: for p‐PI3K/PI3K, si‐NC vs. si‐TMEM175 (*p* = 0.0151) and si‐TMEM175 vs. si‐TMEM175 + 740Y‐P (*p* = 0.0065); for p‐Akt/Akt, si‐NC vs. si‐TMEM175 (*p* = 0.0211) and si‐TMEM175 vs. si‐TMEM175 + 740Y‐P (*p* = 0.0111). The exact *p*‐values for panel C are: for COX2, si‐NC vs. si‐TMEM175 (*p* = 0.0037) and si‐TMEM175 vs. si‐TMEM175 + 740Y‐P (*p* = 0.0082); for IL‐1β, si‐NC vs. si‐TMEM175 (*p* = 0.0004) and si‐TMEM175 vs. si‐TMEM175 + 740Y‐P (*p* = 0.0015); for iNOS, si‐NC vs. si‐TMEM175 (*p* = 0.0002) and si‐TMEM175 vs. si‐TMEM175 + 740Y‐P (*p* = 0.0002).

To investigate the mechanism by which si‐TMEM175 dampens pro‐inflammatory cytokine production, KEGG pathway enrichment analysis revealed a significant enrichment in the PI3K‐Akt signaling pathway among the differentially expressed genes. (Figure 7A), suggesting its potential involvement in TMEM175‐mediated inflammation. To validate this bioinformatic prediction, we assessed the activation status of the PI3K‐Akt pathway by Western Blot. Consistent with the KEGG results, si‐TMEM175 led to a significant reduction in the phosphorylation levels of key signaling components, including PI3K and Akt, compared to si‐NC (Figure 7B). Furthermore, pharmacological activation of the PI3K pathway using 740Y‐P largely reversed the suppressive effect of si‐TMEM175 on pro‐inflammatory cytokine production (Figure 7C). These data indicate that si‐TMEM175 specifically impairs the activation of the PI3K‐Akt pathway. Collectively, our findings demonstrate that TMEM175 acts as a positive regulator of the PI3K‐Akt signaling cascade, and its knockdown attenuates the expression of pro‐inflammatory factors likely through the inhibition of this pathway.

## 5. Discussion

The current study identifies TMEM175 as a critical regulator of innate immune responses and bacterial clearance during sepsis. While TMEM175’s lysosomal regulatory functions have been extensively characterized in neurons [20–24] and its role in maintaining lysosomal pH homeostasis as a proton‐activated proton channel has been validated in COS‐1 cells [20], its expression pattern and immunomodulatory functions in macrophages remain poorly understood. Through scRNA‐seq analysis, we demonstrated that TMEM175 expression was significantly downregulated in monocytes derived from septic patients, with this reduction predominantly occurring in classical monocytes—the subset that serves as a primary precursor of tissue‐infiltrating macrophages. Consistently, across multiple murine sepsis models, macrophage TMEM175 expression was markedly reduced, indicating dynamic regulation under infectious stress. Using adoptive transfer of TMEM175‐overexpressing iBMDM, we found that macrophage‐derived TMEM175 significantly decreased bacterial burdens in the peritoneal cavity and organs of septic mice. Conversely, pharmacological inhibition of TMEM175 with 2‐PPA exacerbated mortality and increased bacterial loads in both compartments. Collectively, these findings confirm that macrophage TMEM175 is required for host defense against invading pathogens.

Both in vitro and in vivo experiments demonstrated that TMEM175 is essential for intracellular bacterial killing but does not affect the phagocytic capacity of macrophages, indicating that TMEM175 specifically regulates post‐phagocytic antimicrobial mechanisms. As central executors of innate immune defense, macrophages eliminate invading pathogens through a tightly orchestrated phagolysosomal pathway [8]. This process begins with pathogen recognition by pattern recognition receptors (e.g., TLRs and CLRs) [33, 34], followed by internalization into phagosomes that progressively mature into bactericidal phagolysosomes [13]. The acidic luminal environment (pH 4.5–5.0) of phagolysosomes is critical for optimal activation of hydrolytic enzymes and serves as a key regulatory node for antimicrobial defense [6, 17, 18]. TMEM175 was previously defined as a lysosomal K^+^ channel under nonphysiological, neutral pH conditions that do not reflect the environment of healthy lysosomes [22]. Furthermore, Hu et al. [20] identified TMEM175 as a proton‐activated proton channel that maintains lysosomal pH homeostasis under physiological conditions. They demonstrated that proton flux through TMEM175 is required for maximal lysosomal hydrolase activity and that TMEM175 deficiency‐induced hyper‐acidification impairs enzymatic function and promotes α‐synuclein aggregation in vivo [20, 35]. All these lysosomal hydrolases, such as cathepsin B and CTSD, are indispensable for pathogen degradation [14, 15, 36]. While TMEM175 deletion has been reported to cause lysosomal over‐acidification and impair proteolytic activity [20], our findings extend the prevailing model that attributes decreased hydrolase activity solely to the altered pH. Here, we provide evidence that TMEM175 regulates hydrolase expression at the transcriptional level, beyond merely modulating lysosomal acidification. These data uncover a previously unrecognized transcriptional regulatory function of TMEM175 in antimicrobial immunity.

It is worth noting that the optimal luminal pH of lysosomes is 4.5–5.0. Although *TMEM175* KO lysosomes exhibit an approximate 0.4 pH unit of over‐acidification [20], the enhanced acidity is unlikely to be the primary driver of impaired CTSD activity. This conclusion is supported by the observation that CTSD retains ≥80% of its maximal activity across the broad pH range of 3.5–5.0 [37, 38]. Our RNA‐seq analysis further revealed that TMEM175 knockdown markedly repressed the transcription of multiple CTS‐family genes, including CTSD, CTSE, CTSF, CTSS, and CTSH. Collectively, these data indicate that TMEM175 controls mature cathepsins at the transcriptional and post‐transcriptional levels [39]. In multiple lines of TMEM175 KO cells (BMDM and iBMDM), cathepsin maturation was significantly impaired, a phenomenon directly attributable to a marked reduction in cathepsin mRNA expression. This transcriptional deficit, rather than lysosomal hyper‐acidification, accounted for the decreased steady‐state levels of mature cathepsins, which in turn impaired intracellular bacterial killing.

Beyond lysosomal proteolysis, TMEM175 deficiency led to broader organelle dysfunction. We observed a significant loss of mitochondrial membrane potential and a secondary late‐stage blockade in autophagic flux, as evidenced by altered LC3B‐II/I ratios and p62 accumulation. These findings are consistent with the notion that lysosomal dysfunction disrupts the autophagic clearance of damaged mitochondria [21, 31]. Notably, TMEM175‐knockdown macrophages exhibited markedly elevated intracellular ROS levels; however, NAC‐mediated ROS neutralization failed to restore mature CTSD levels and paradoxically exacerbated intracellular bacterial survival. This unexpected result suggests that in the context of TMEM175 deficiency, the elevated ROS serve as a compensatory antimicrobial mechanism rather than a pathogenic mediator of the bactericidal defect—a finding consistent with the established role of the oxidative burst in macrophage‐mediated bacterial killing [12, 32]. These observations extend the functional consequences of TMEM175 loss beyond lysosomal proteolysis to encompass mitochondrial quality control and redox homeostasis, further underscoring the multifaceted role of TMEM175 in maintaining macrophage antimicrobial competence.

Currently, the clinical treatment of sepsis is still based on broad‐spectrum antibiotic therapy targeting the presumed pathogen, combined with supportive interventions to stabilize hemodynamics and metabolic homeostasis [40, 41]. However, with the increasing understanding of the limitations of antibiotic resistance and the pathophysiological complexity of sepsis, which is characterized by the simultaneous presence of excessive inflammation and immune paralysis, there has been a strong interest in immunomodulatory strategies [42, 43]. We found that knockdown of TMEM175 led to the downregulation of inflammatory cytokine expression in macrophages through the PI3K‐Akt pathway. The simultaneous inhibition of inflammatory cytokine production in TMEM175‐deficient macrophages revealed its broader immunomodulatory role. Although excessive inflammation contributes to late sepsis pathology, early cytokine signaling is crucial for pathogen control [42]. Therefore, the disruption of this critical balance by TMEM175 deficiency impairs the host defense. Our findings extend the known neuronal functions of TMEM175 to bacterial infection‐induced systemic inflammation, revealing its critical role in regulating both bacterial clearance and inflammatory responses within macrophages. Mechanistically, TMEM175 mediates these effects through two complementary pathways: both enhancing pathogen clearance via direct transcriptional regulation of cathepsins and lysosomal pH‐dependent maturation of these proteases and promoting the release of inflammatory cytokines. Notably, the direct transcriptional regulation of CTSD appears to represent the primary mechanism, while lysosomal pH modulation serves as a downstream effector that synergistically enhances the efficacy of pathogen clearance. In parallel, TMEM175 in macrophages promotes the expression of pro‐inflammatory cytokines, which may contribute to the recruitment and activation of neutrophils, potentially exacerbating inflammatory responses. Collectively, these dual functions of TMEM175 highlight its potential as a promising therapeutic target in the management of sepsis, particularly in clinical contexts requiring precise regulation of immune activation and pathogen clearance.

Although all patients met the Sepsis‐3 criteria, these criteria do not fully capture differences in disease severity, infection source, sampling time, treatment, or comorbidities, all of which may influence monocyte composition and TMEM175 expression. Future studies with detailed clinical annotation and appropriate stratified analyses will therefore be needed to determine how these factors contribute to the variation observed among patients. In parallel, measuring the relative abundance of classical, intermediate, and nonclassical monocytes and examining TMEM175 expression within each subset would clarify whether this variation arises from changes in the monocyte‐subset composition or from altered TMEM175 expression within individual subsets. This approach is supported by previous single‐cell evidence showing substantial monocyte heterogeneity in sepsis [44]. Age may represent another source of variation as previous analyses have shown that the transcriptional response to sepsis differs among age groups [45]. If future PBMC RNA‐seq analyses include pediatric (<18 years), adult (18–64 years), older (65–80 years), and oldest‐old (>80 years) patients together with age‐matched controls, we would expect TMEM175 expression to remain lower in patients with sepsis across these groups. However, the extent of this reduction and the associated transcriptional programs may differ with age, with pediatric patients potentially displaying a response distinct from that of adults. Because bulk PBMC RNA‐seq reflects the combined signals of multiple immune cell populations, changes in cell composition should also be considered when interpreting these age‐related differences.

Overall, our findings challenge the prevailing view that TMEM175 deficiency promotes Parkinson’s disease primarily by driving lysosomal hyper‐acidification. Although widely cited [20, 35], the experimental evidence supporting this hyper‐acidification mechanism remains insufficient to support a definitive conclusion. In contrast, we demonstrate that TMEM175 is required for the transcriptional regulation of multiple cathepsins during bacterial infection, a pathway indispensable for an effective innate immune response and for survival from sepsis. By establishing TMEM175 as a critical regulator of cathepsin expression rather than a mere lysosomal proton leak channel, our work extends the therapeutic scope of TMEM175 to restore dysregulated host defense.

## Author Contributions

Zhenzhen Bai, Tao Luo, and Lingbin Sun contributed to the conception of the study. Zhenzhen Bai and Bei Peng performed the experiments. Zhenzhen Bai analyzed data and wrote the manuscript. Lingbin Sun confirm the authenticity of all the raw data.

## Funding

This work was supported by the Natural Science Foundation of Guangdong Province, Grant 2024A1515030274; the Shenzhen Science and Technology Foundation, Grant JCYJ20250604191315021; the Shenzhen Sanming Project, Grant SZSM202211040; and the Guangdong Basic and Applied Basic Research Foundation, Grant 2025A1515220037.

## Disclosure

All authors have read and approved the final manuscript.

## Conflicts of Interest

The authors declare no conflicts of interest.

## Supporting Information

Additional supporting information can be found online in the Supporting Information section.

## Supporting information


**Supporting Information** Supporting Information: The supporting information for this article includes the following: Supporting Figures: A document containing Figure S1: (Sub‐clustering analysis of monocytes) and Figure S2: (Flow cytometry for macrophage purity). Supporting File 1: A document containing Table S1: (Characteristics of septic patients and healthy controls), Table S2: (Primer sequences for real‐time PCR), and Table S3: (Details of primary antibodies used in this study).

## Data Availability

The data that support the findings of this study are available from the corresponding author upon reasonable request.
