# Supplementary material for: Macrophage TMEM175‐Mediated Transcriptional Regulation of Cathepsins Drives Bacterial Killing in Sepsis
Source: J Immunol Res. 2026 Sep 18;2026:2046607. doi: 10.1155/jimr/2046607 (PMC13587704; doi:10.1155/jimr/2046607)
Supplement: Supplementary file 1 [file JIMR-2026-2046607-s001.zip › Supplementary figures.docx]

Supplementary Figure 1

Supplementary Figure 1. Stacked bar chart illustrating the proportional composition of three monocyte subtypes (classical, intermediate, non-classical) in individual healthy controls (HC) and septic patients. Each vertical bar represents one independent biological donor.

Supplementary Figure 2

Supplementary Figure 2. Flow cytometry gating strategy for human peripheral blood monocytes. Representative dot plot showing monocyte identification via CD11B and LY6G staining; Q1–Q4 quadrant proportions are labeled on the plot.
