# Supplementary material for: Macrophage TMEM175‐Mediated Transcriptional Regulation of Cathepsins Drives Bacterial Killing in Sepsis
Source: J Immunol Res. 2026 Sep 18;2026:2046607. doi: 10.1155/jimr/2046607 (PMC13587704; doi:10.1155/jimr/2046607)
Supplement: Supplementary file 1 [file JIMR-2026-2046607-s001.zip › Supplementary File 1.docx]

**Table S1.** Characteristics of septic patients and healthy control.

| Characteristics | Gram-negative sepsis  (n = 44) |  | | Healthy control  (n=44) | |  | |  |
| --- | --- | --- | --- | --- | --- | --- | --- | --- |
| Age (yr) | 61.89 ±15.42 | |  | | 61.09 ± 12.35 | |  | |
| Sex, male (%) | 29 (65.91%) | |  | | 29 (65.91%) | |  | |
| APACHE-Ⅱ | 24.52 ±8.17 | |  | |  | |  | |
| SOFA | 6.73 ±3.88 | |  | |  | |  | |
| Sepsis due to |  | |  | |  | |  | |
| Peritonitis | 10 (20.41%) | |  | |  | |  | |
| Multiple injuries | 7 (14.29%) | |  | |  | |  | |
| Urinary tract infections | 6 (12.24%) | |  | |  | |  | |
| Pneumonia | 23 (46.94 %) | |  | |  | |  | |
| Other infections | 3 (6.12 %) | |  | |  | |  | |
| Length of ICU stay | 12.09 ±11.70 | |  | |  | |  | |
| 28-day mortality | 12 (27.27%) | |  | |  | |  | |

Data are expressed as the mean ± SD or number (%) where applicable. Multi-group comparisons were assessed using ANOVA test. Differences in categorical data were compared using Fisher exact test or Chi-Squared test.

ICU = Intensive Care Unit; APACHE II = Acute Physiology and Chronic Health Evaluation II; SOFA = Sequential Organ Failure Assessment.

**Table S2.** Primer sequences for real-time PCR.

| Gene | Sequence (from 5' to 3') |
| --- | --- |
| human *β*-actin | GGCACCCAGCACAATGAA |
|  | TAGAAGCATTTGCGGTGG |
| human TMEM175 | GTGGGCTATGCCTTCCACTT |
|  | AGACAGCCGCAACAAAACAC |
| mouse *β*-actin | TCATCACTATTGGCAACGAGC  AACAGTCCGCCTAGAAGCAC |
| mouse TMEM175 | GTGGGCTATGCCTTCCACTT  AGACAGCCGCAACAAAACAC |
| Cathepsin D | ATCCTCAAAGGCCCCATCAC  GGGACCCACAGGTTAGAGGA |
| Cathepsin E | AGTGGATTGTGCCACTCTCG  AAAGCCACTGCCACAGAACT |
| Cathepsin F | ACCTTGCAATGATCCCCTGG  CCTTGGCATTCACTGGGCTA |
| Cathepsin H | AAAAATCAGGGGGCCTGTGG  ACCTCCTTTGCAGCCATGAT |
| Cathepsin S | GTGACGAGGATGCCCTGAAA  GGTCGTCATAGACACCGCTT |

**Table S3.** Primary antibodies

| Primary antibodies | Manufacturer | Model Number |
| --- | --- | --- |
| anti-TMEM175 | Proteintech | 68172 |
| anti-Cathepsin D | Abcam | ab302649 |
| anti-LAMP-1 | ABclonal | A16894 |
| anti-COX2 | ABclonal | A1253 |
| anti-iNOS | ABclonal | A18247 |
| anti-*β*-actin | ABclonal | AC026 |
| anti-IL-1β | Abcam | ab234437 |
| anti-Akt | Cell Signaling Technology | 4691 |
| anti-phospho- Akt | Cell Signaling Technology | 4060 |
| anti-PI3K | ABclonal | A4992 |
| anti-phospho- PI3K | Cell Signaling Technology | 17366 |
